# Adaptive Evolution of the STRA6 Genes in Mammalian

**DOI:** 10.1371/journal.pone.0108388

**Published:** 2014-09-24

**Authors:** Jianghong Wu, Hui Xiang, Yunxia Qi, Ding Yang, Xiaojuan Wang, Hailian Sun, Feng Wang, Bin Liu

**Affiliations:** 1 Inner Mongolia Prataculture Research Center, Chinese Academy of Science, Hohhot, China; 2 Animal Husbandry Institute, Inner Mongolia Academy of Agricultural & Animal Husbandry Sciences, Hohhot, China; 3 Kunming Institute of Zoology, Chinese Academy of Sciences, Kunming, China; Federal University of Rio de Janeiro, Brazil

## Abstract

Stimulated by retinoic acid 6 (STRA6) is the receptor for retinol binding protein and is relevant for the transport of retinol to specific sites such as the eye. The adaptive evolution mechanism that vertebrates have occupied nearly every habitat available on earth and adopted various lifestyles associated with different light conditions and visual challenges, as well as their role in development and adaptation is thus far unknown. In this work, we have investigated different aspects of vertebrate *STRA6* evolution and used molecular evolutionary analyses to detect evidence of vertebrate adaptation to the lightless habitat. Free-ratio model revealed significant rate shifts immediately after the species divergence. The amino acid sites detected to be under positive selection are within the extracellular loops of STRA6 protein. Branch-site model A test revealed that *STRA6* has undergone positive selection in the different phyla of mammalian except for the branch of rodent. The results suggest that interactions between different light environments and host may be driving adaptive change in STRA6 by competition between species. In support of this, we found that altered functional constraints may take place at some amino acid residues after speciation. We suggest that *STRA6* has undergone adaptive evolution in different branch of vertebrate relation to habitat environment.

## Introduction

During the evolution of animal kingdom, sunlight has been the most potent selective force to control the evolution of living organisms [1].The evolution of photo-detection, giving rise to eyes, offers a kaleido-scopic view of selection acting at both the organ and molecular levels [2]. The eye employs remarkable adaptations to environmental challenges. The gradual evolution of novel molecular/developmental mechanisms in eye and vision allow animals to live in different light environments (aquatic/terrestrial/subterranean/nocturnal) [3]–[5]. Despite the enormous morphological variations found among animal eyes [1], genetic studies have indicated that all eyes may share a similar developmental cascade of transcription factors [6]. The ease with which we can now analyze the evolution of structural gene sequences across species belies the difficulties in tracing the selective forces that shaped regulation of gene expression [2].

Vertebrate vision depends on light-dependent isomerization of a chromophore (11-cis-retinal) bound to the visual pigment opsin triggering the phototransduction cascade, and resulting in neural signals being sent to the brain. While the light-dependent reaction occurs in the photoreceptor cells, the enzymatic trans-to-cis re-isomerization occurs in the cells of the retinal pigment epithelium (RPE), a monolayer epithelium adjacent to and partly enclosing the photoreceptor cells. In the vertebrate eye, all-trans-retinol bound to RBP (holo-RBP) is delivered to the RPE through choriocapillaris blood. The holo-RBP interacts with the basal side of RPE cells via STRA6 protein [7]. STRA6 is a cell-surface receptor for retinol-RBP that removes retinol from RBP and transports it across the plasma membrane [8].

*STRA6* was initially isolated as an RA-inducible gene in P19 embryonal carcinoma cells [9]. Human *STRA6* protein contains nine potential transmembrane domains [10]. *STRA6* of cow has eleven putative transmembrane domains [7]. Then, by inserting an epitope tag into all possible extracellular and intracellular domains of *STRA6*, Kawaguchi and colleagues find *STRA6* has 19 distinct domains, including five extracellular domains, nine transmembrane domains, and five intracellular domains. *STRA6* is a multi-transmembrane domain protein, as a specific membrane receptor for retinoid binding protein (RBP) [11]. *STRA6* binds to RBP with high affinity and has robust vitamin A uptake activity from the vitamin A-RBP complex. *STRA6* not only is a vitamin A transporter but also is a cell-surface signaling receptor activated by the RBP–retinol complex [12].Mutations in *STRA6* cause a broad spectrum of malformations including anophthalmia, congenital heart defects, diaphragmatic hernia, alveolar capillary dysplasia, lung hypoplasia, and mental retardation in human [13]. In *STRA6* ^−/−^ null mice, rod photoreceptor outer and inner segment length was reduced, and cone cell numbers were reduced, as were scotopic and photopic responses [14]. *STRA6* also was required for dissolution of the primary vitreous. Moreover, some studies have suggested that *STRA6* mutation can cause isolated eye malformations in addition to the congenital anomalies observed in Matthew-Wood syndrome [15].

In this study, we hypothesize that *STRA6* may be a critical determinant of the eyes and vision across vertebrates. Here we use a molecular evolution approach to test whether key evolutionary transitions in the eye or vision of vertebrates have involved the *STRA6*. We conduct a wide survey of vertebrates, including representatives from *Actinopterygii*, *Amphibian*, *Reptilian*, *Aves* and *Mammalian*, and apply codon substitution models to identify lineages and sites under Darwinian selection.

## Materials and Methods

### Sequence acquisition

Sixty-eight *STRA6* sequences, respectively from *Rattus norvegicus, Danio rerio, Bos taurus, Pongo pygmaeus abelii, Homo sapiens, Mus musculus, Orcinus orca, Trichechus manatus latirostris, Odobenus rosmarus, Ochotona princeps, Sorex_araneus, Octodon degus, Jaculus jaculus, Condylura cristata, Echinops telfairi, Anas platyrhynchos, Ficedula albicollis, Mesocricetus auratus, Melopsittacus undulatus, Falco peregrinus, Chrysemys picta bellii, Microtus ochrogaster, Chinchilla lanigera, Geospiza fortis, Falco cherrug, Columba livia, Pantholops hodgsonii, Alligator sinensis, Bubalus bubalis, Tupaia chinensis, Camelus ferus, Vicugna pacos, Alligator mississippiensis, Myotis davidii, Chrysochloris asiatica, Elephantulus edwardii, Pteropus alecto, Peromyscus maniculatus bairdii, Chelonia mydas, Panthera tigris altaica, Physeter catodon, Balaenoptera acutorostrata scammoni, Python bivittatus, Lipotes vexillifer, Cricetulus griseus, Ornithorhynchus anatinus, Callorhinchus milii, Orycteropus afer, Stegastes partitus, Macaca mulatta, Xenopus tropicalis, Otolemur garnettii, Pan_paniscus, Pan paniscus, Papio anubis, Felis catus, Ovis aries, Gorilla gorilla, Tursiops truncatus, Ceratotherium simum simum, Dasypus novemcinctus, Heterocephalus glaber, Erinaceus_europaeus, Chlorocebus sabaeus, Eptesicus fuscus, Gallus gallus, Pan troglodytes, Canis familiaris* were obtained from Genbank. We also obtained Ensembl predicted STRA6 sequences from published genomes(http://www.ensembl.org) for *Ailuropoda melanoleuca, Cavia porcellus, Equus caballus, Gasterosteus aculeatus, Latimeria menadoensis, Loxodonta Africana, Meleagris gallopavo, Myotis lucifugus, Monodelphis domestica guttatus, Mustela putorius furo, Oryctolagus cuniculus, Galago senegalensis, Oreochromis niloticus, Petromyzon marinus, Pelodiscus sinensis, Pteropus vampyrus, Sus scrofa, Tetraodon nigroviridis, Takifugu rubripes, Xiphophorus maculatus* (File S1).

### Sequence alignment and phylogenetic analyses

**Figure 1:**
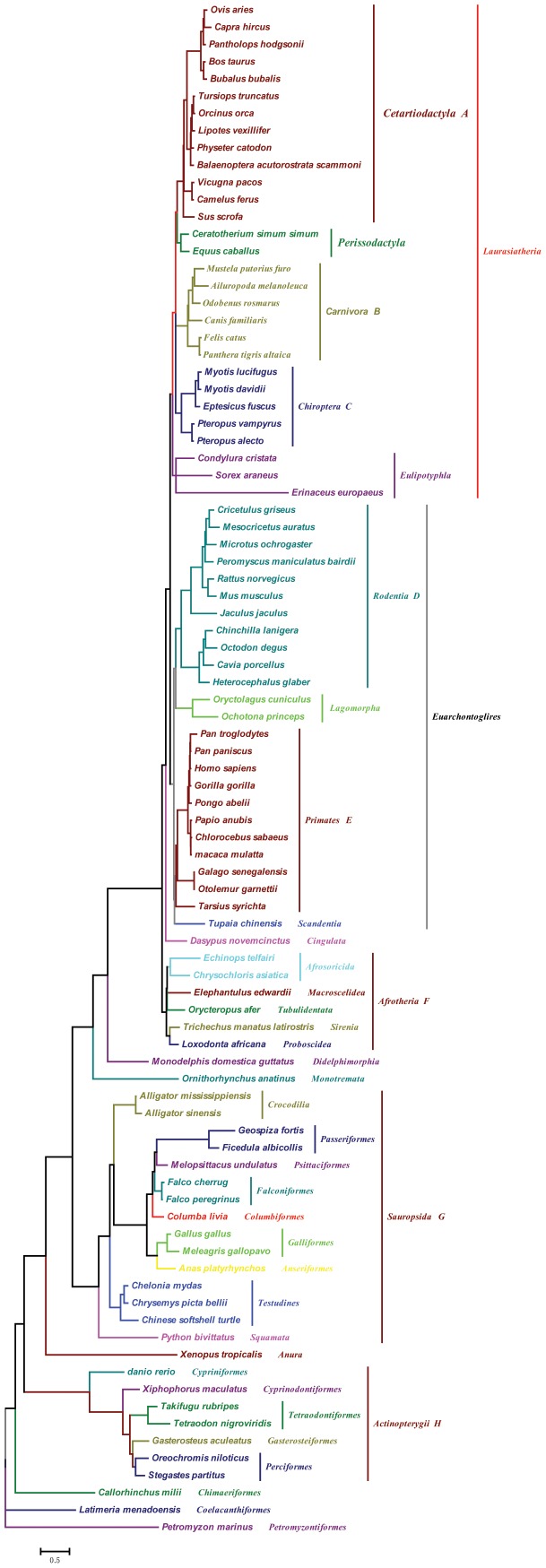
Phylogenetic tree of vertebrates studied in this paper. The topology is based on publication. Eight branches tested both by the two-ratio model tests and the branch-site model A tests were marked by A, B, C, D, E, F, G and H, respectively.

In total, 89 species of vertebrates were studied and a phylogenetic tree was constructed based on *STRA6* gene sequence. These species belonged to 7 classes and 35 orders were used for the evolutionary analysis of *STRA6*. Multiple sequence alignments were performed ClustalW [16] and MEGA 6 [17]. A vertebrate species tree used for the evolutionary analysis was constructed according to the topology of previous publications [18]–[22] (Fig. 1).

### Molecular Evolution Analyses

To test for evidence of molecular adaptation in *STRA6*, we used the CODEML program in PAML 4 to derive ML estimates of the rates of synonymous and nonsynonymous substitutions (dS and dN, respectively) and the dN/dS ratio (omega, ω) [23]. The ω ratio is a measure of natural selection acting on the protein. Simplistically, values of ω<1,  = 1, and >1 means negative purifying selection, neutral evolution, and positive selection. The topology of which was constrained based on published studies and is shown as Fig. 1. For all PAML-based analyses, alignment gaps were treated as ambiguity characters (setting cleandata = 1). Branch lengths were estimated simultaneously (iteration setting method = 0), and codon frequencies were calculated from the average nucleotide frequencies at the three codon positions (setting CodonFreq = 2 (F3X4)).

We first tested whether the gene shows evidence of diverse selective pressures across the species by implementing the free-ratio model, comparing this to a null model in which all branches have a uniform selective pressure (one-ratio model). To test selective pressure of the *STRA6* genes in different vertebrate lineages, we performed two-ratio model and compared it with one-ratio model through a likelihood ratio test (LRT). The two-ratio model assumes focal lineage(s) have a different ω value than others lineages; whereas, the one-ratio model supposes ω value is the same across all branches. The statistic 2Δ*l* (twice the log likelihood difference between the nested models) was compared with the chi-square distribution. First, we tested the ancestors leading to *Chiroptera*, *Actinopterygii*, *mammalian*, *rodent*, *sauropsida* and *terrestrial* animal individually. Then we tested whether the ω value in six ancestral branches was significantly higher than background lineages. In all cases, the one-ratio model, which assumes the equal dN/dS ratio among all branches, was performed as a null model.

To identify the sites under positive selection along the *STRA6* genes, we implemented site models in which ω can vary among sites. “Site-specific” models allowing ω = dN/dS, to vary among sites were used to detect candidate positively selected sites in the STRA6 gene [24]. This survey was achieved by three comparisons among four models: M2a vs. M1a, and M8 vs. M7 [23]. Significant difference between the two models was accepted if this difference was greater than twice the average log-likelihood difference between the models, a distribution that follows a χ^2^ distribution. When the likelihood ratio tests indicated significance, the Bayes empirical Bayes method was used to calculate posterior probabilities for the potential positively selected sites [24]. Furthermore, the dN/dS values on the different lineages were evaluated using a free-ratio model compared with a one-ratio model where all lineages have a single dN/dS value with degrees of freedom (*df*) equal to the difference in the number of parameters of the two models.

### Mapping Residues Evolving under Positive Selection on the Structures

To infer structure–function correlations, amino acid residues on which positive Darwinian selection has been operating were mapped onto the structure of the STRA6 (modeled as in [25]. Unfortunately, the three-dimensional (3D) structure of STRA6 is not available in the Protein Data Bank (http://www.rcsb.org/pdb). Here, high-resolution de novo 3D structure of STRA6 was predicted by ROBETTA http://robetta.bakerlab.org/ [26]. Then, using RASMOL [27] mapped residues evolving under positive selection in 3D structure of STRA6.

## Results

### Alignment of amino acids for STRA6 protein

Alignment of the STRA6 protein sequence across 89 vertebrate species showed that a 38 residues long segment was present in ferret and 11 or 12 amino acids in other species. This range is seated at residues 74 and 87 of the human STRA6 protein, which is located in first intracellular loops of STRA6 protein. Our analysis provides an alternative avenue to explain the less well visual system of ferret (File S2).

### Variation in the rate of molecular evolution over STRA6

**Figure 2:**
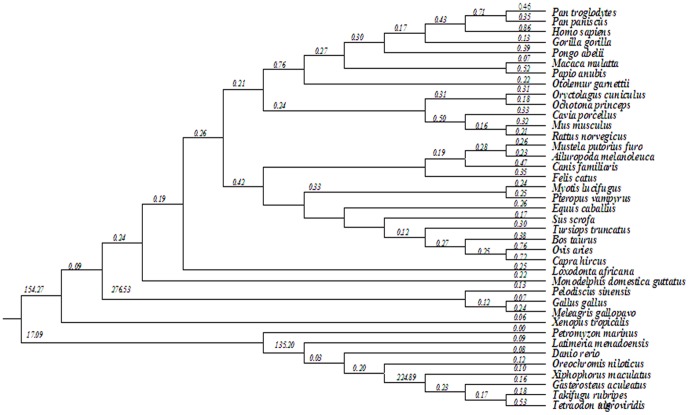
ω Values for distinct evolutionary lineages of vertebrate, with a phylogenetic tree derived from fig. 1. The ω values for individual branch according to the free-ratio model are shown.

The results obtained from the one-ratio model indicated that the vertebrate was under purifying selection (ω = 0.243). Each vertebrate lineage was specified as a foreground branch to test for adaptive selection separately in each derived lineage. Results of all two-ratio model tests for seven branches (branch A–H, Fig. 1) are shown in Table 1. Our results showed that the *Primates*, *Chiroptera*, *Carnivora*, *Rodent*, *Cetartiodactyla* and *Afrotheria* branch had significantly higher ω value compared with other branches, suggesting the potential action of positive selection during early stages of the evolution of six mammalian phyla. However, the two-ratio model which designed the ancestral branch of *Sauropsida* and *Actinopterygii* (the branch marked with G and H in Fig. 1) as foreground was a significantly better fit to the dataset than the one-ratio model (both P = 0) (Table 1). The estimated ω value on the ancestral branch of *Sauropsida* and *Actinopterygii* were lineages of magnitude lower than that of background (0.19305 vs. 0.25688 and 0.17541 vs. 0.25236, respectively, Table 1). These results indicating a selection pressure change acting on *STRA6* in the vertebrates. Hence, we used the free-ratio model of substitution rates that assigns a different rate to each branch of the tree had a significantly better fit than a model with a single rate for all vertebrates branches (table 1; *χ* ^2^ = 544.66, P = 0 with *df* = 174). The results from free-ratio model showed *STRA6* genes were under different selective pressures in vertebrates with a suggestion of higher ωvalues in branch of *Cetartiodactyla, Primates, Afrotheria and Sauropsida* (Fig. 2).

**Table 1:** Results of two-ratio model tests of selection pressure on the STRA6 gene in vertebrate.

Model	Np	*l*	*ω*_0_ *^a^*	*ω^a^*	Model Compared	2Δ*l*	*P*
**A.** One ratio: ***ω*** **_0_**	*177*	*−75502.66*	0.24280	= ***ω*** **_0_**			
**B.** Free ratio	*351*	*−75230.33*			**B**_VS._ **A**	544.66	0
**C.** Two ratios: ω_0_, ω_A_	178	−75492.31	0.23709	0.32960	**C**_VS._ **A**	*20.7*	0.0000
**D.** Two ratios: ω_0_, ω_B_	178	−75486.85	0.23622	0.36393	**E**_VS._ **A**	*31.62*	0
**E.** Two ratios: ω_0_, ω_C_	178	−75500.15	0.24090	0.30001	**F**_VS._ **A**	*5.02*	0.0250
**F.** Two ratios: ω_0_, ω_D_	178	−75499.26	0.23764	0.26994	**H**_VS._ **A**	*6.8*	0.0091
**G.** Two ratios: ω_0_, ω_E_	178	−75499.95	*0.24034*	*0.29153*	**J**_VS._ **A**	*5.42*	0.0199
**H.** Two ratios: ω_0_, ω_F_	178	−75500.30	*0.23986*	*0.27722*	**K**_VS._ **A**	*4.72*	0.0298
**I.** Two ratios: ω_0_, ω_G_	178	−75483.61	*0.25688*	*0.19305*	**M**_VS._ **A**	*38.1*	0
**J.** Two ratios: ω_0_, ω_H_	178	−75484.24	*0.25236*	*0.17541*	**N**_VS._ **A**	*36.84*	0

*a ω*(ω_A_, ω_B_, ω_C_, ω_D_, ω_E_, ω_F_, ω_G_, ω_H_) and ω_0_, are the ratios for branches A, B, C, D, E, F, G, H and other branches, respectively (see Fig. 1).

Then we performed the test 2 of branch-site model A to detect the positively selected sites on the above seven ancestral branches leading to different lineages of vertebrates. Results of all tests 2 of the branch-site model A are shown in File S3. The positive selection was detected on the branches of leading to *Primates*, *Chiroptera*, *Carnivora*, *Cetartiodactyla* and *Afrotheria* (branch A,B,C,E,F in Fig. 1). Statistically supported evidence of positive selection was detected on these ancestral branches.

### Positive selection in the STRA6 gene

To further investigate the possibility of positive selection, we performed a maximum-likelihood-based analysis of codon substitution models. The parameters of four models of coding sequence evolution, taking into account the variation of *ω*, were estimated (Table 2). We tested the hypothesis of positive selection against purifying selection with a LRT (M8 versus M7 model). The results show that the LRT between model M7 and model M8 is significant (2*Δl* = 58.42, *df* = 2, *P* = 0), suggesting that model M8 was better than model M7, and five sites was identified as being under positive selection. Altogether, these models found some positively selected sites.

**Table 2:** Models of variation of the *STRA6* sequence in 39 vertebrates.

Model	*np*	*lnL* a	Estimates of parameters^b^	Positively selected sites^c^
Free-ratio	351	−75230.33	*k* = 2.94205	
M7 (beta)	178	−75502.66	*k* = 2.98981	Not allowed
			*p* = 0.78545 *q* = 2.09076	
M8 (beta and ω)	180	−75473.45	*k* = 3.02332	**175P**,**176E**,**177T**,**178S**,**181R**
			*P*_0_ = 0.98956 (*P*_1_ = 0.82695)	
			*p* = 0.01064 *q* = 2.39217	
			*ω* = 4.23045	

a *Log-likelihood value.*

b *k estimate of transition/transversion rate ratio.*

c *Positive selection sites are identified at the cutoff p>95%, with those with 99% shown in boldface.*

### Mapping Residues Evolving under Positive Selection on the Structures

**Figure 3:**
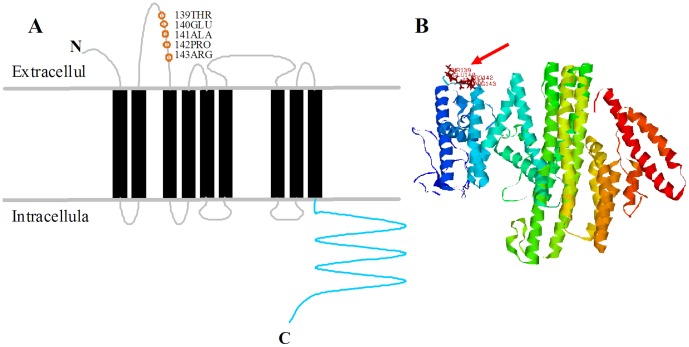
Mapping of residues evolving under positive selection onto the structure of STRA6 protein. (A) Schematic protein model of STRA6 was based on [25]. The positive selection amino acids are indicated with orange circles, the nine transmembrane domains are indicated with black bars, and the intracellular membrane proximal region is indicated by a green dotted line. (B) Mapping of residues evolving under positive selection onto 3D structure. The residues evolving under positive selection locate in coil of 3D structure, which take place in the membrane proximal region (indicated by the red arrow).

To infer structure–function correlations, amino acid residues on which positive Darwinian selection has been operating were mapped onto the structure of the STRA6. Since the 3D structure for the Human STRA6 protein has been predicted by ROBETTA, it is possible to map these positions in 3D space. Five amino acid positions predicted to be under selection by maximum likelihood are located on the coil between two transmembrane domains of STRA6 protein. These 5 residues (175P, 176E, 177T, 178S and 181R) corresponds to human STRA6 amino acid (139THR, 140GLU, 141ALA, 142PRO and 143ARG), because many gaps are exist in alignment of *STRA6* gene sequences (Fig. 3). The results show that the five residues locate at extracellular, which may play an essential role to binding holo-RBP. However, the selective pressure producing the signal of positive selection demonstrated here remains unknown.

## Discussion

The increasing availability of genomic sequence data and high throughout annotation of genes from a wide range of animal taxa enables bioinformatics analysis of genes of interest and to provide important insight into their evolutionary link with particular phenotypic trait and association with human disease. Mutation *STRA6* gene have been reported to cause eye malformations in human [13] and reduce rod photoreceptor outer and inner segment length and cone cell numbers in mice [14]. Biochemical and genetic studies have confirmed the STRA6 protein is a vitamin A transporter and essential for the vision responses [7], [28]. Thus, we hypothesized that *STRA6* gene play an important role in visual variation in vertebrates. To test this hypothesis, we used the ML estimates of the rates of synonymous and nonsynonymous substitutions.

Our molecular evolutionary analyses of vertebrate *STRA6* revealed interesting and distinct patterns of selection in the data sets. Here, we summarize our findings in light of past studies of STRA6 biochemistry and speculate on the possible adaptive relevance of sequence variation in vertebrates. The results obtained from the One-ratio model indicated that STRA6 gene was under purifying selection in most vertebrates and suggested that this gene is functionally important. In addition, our free-ratio model evolutionary studies revealed that a selection pressure change acting on STRA6 in the vertebrates. The ω ratio significantly higher than one is convincing evidence for diversifying selection [29]. Nonfunctional visual genes are usually associated with species that inhabit poor light environments (aquatic/subterranean/nocturnal), and these genes are believed to have lost function through relaxed selection acting on the visual system [30].

On the other side, early eutherian mammals faced competition with diurnal reptiles (e.g. dinosaurs) during the Mesozoic era according the nocturnal bottleneck hypothesis [31]. We suggest that these elevated ω values in some branches of *mammalian* is probably related to the evolution of the eye and vision in these taxa that is needed for their observation after the Mesozoic era. Our results show that four episodes of higher ω values in *Primates*. Jacobs present a view that the main evolutionary trend for the primate branch was adaptation to diurnality [32]. Boris Joffe and colleagues show that transition to diurnality occurred independently in several primate and related groups:Tupaia, diurnal lemurs (independently in at least two families), and, at least partially independently, in Simiiformes (monkeys and apes) and Tarsiiformes [33]. Our findings are consistent with the evolution event of primates that Boris Joffe presented. Interestingly, previous work on Prestin gene [34] and Cldn14 gene [35] showed positive selection at the same evolutionary stages of cetacean evolution as for STRA6; that is, the ancestor of toothed whales. Thus, the co-timing of positive selection in these three genes offers strong evidence of molecular adaptive evolution in early whales. STRA6 may be an important gene for the evolution of eutherian eye and vision. Moreover, other vertebrate lineage showed ω value larger than one, including *Afrotheria* and *Sauropsida*. These findings provide more evidence that the STRA6 gene might be important for the visual evolution of vertebrates.

Results from two-ratio model tests confirmed the free-ratio result, and showed that selection pressure change was found in major focal branches of vertebrates, such as *Primates*, *Rodents*, *Carnivora*, *Chiroptera*, *Cetartiodactyla*, *Afrotheria*, *Actinopterygii* and *Sauropsida*. The two-ratio model suggested that the mammlian had a significantly higher ω ratio than non-mammlian. It suggests that the evolution of the STRA6 gene of mammalian is accelerated evolutionary compare with other vertebrate branches after the Mesozoic era.

Apart from branch model, we also tested these eight branches using branch-site model. The results revealed that *STRA6* has undergone positive selection in the branch of *Primates*, *Carnivora*, *Chiroptera*, *Cetartiodactyla*, and *Afrotheria*. However, Rodent, *Sauropsida and Actinopterygii* showed no amino acid sites identified as being under positive selection. In the absence of positive selection, one possibility is that Rodents have experienced relaxed selection, perhaps due to a relatively higher dependence on the vision system. Among mammals, such UV-sensitive visual pigments are relatively rare and have only been described in some rodents. These finding suggested that the evolutional pattern for eye and vision of rodents may be different to other mammalian after the dinosaurs become extinct.

Site-specific models Analyses of the *STRA6* gene showed five sites were under positive selection. All of them were located in extracellular loops of the STRA6 protein structure. STRA6 exhibit a common architecture of nine transmembrane helices (TMs) linked by intracellular loops and extracellular loops (ECLs). Extracellular loops was an essential domain for RBP binding [36]. Researches show that homozygous mutations (P90L, P293L, T321P, T644M and R655C) in STRA6 cause a pleiotropic, multisystem malformation syndrome in human [13]. In the last few years, the less conserved extracellular loops have garnered increasing interest, particularly after the publication of several GPCR crystal structures that clearly show the extracellular loops to be involved in ligand binding [37], [38]. In addition, the ferret STRA6 structures insert a long segment of intracellular loops to change the protein conformation. Our analysis provides an alternative avenue to explain the less well visual system of ferret. Given the fact that, functional shifts has been assigned to even single amino acid replacement during species evolution [39], it is conceivable to argue that five positively selected position provide a set of specific candidates for future functional experiments to elucidate vision adaptation.

## Conclusions

In this study, we have investigated different aspects of vertebrate *STRA6* evolution and used molecular evolutionary analyses to detect evidence of vertebrate adaptation to the lightless habitat. Free-ratio model evolutionary studies revealed that a selection pressure change acting on STRA6 in the vertebrates. The amino acid sites detected to Branch-site model A test revealed that *STRA6* has undergone positive selection in the different phyla of mammalian except for the branch of rodent. The results suggest that interactions between different light environments and host may be driving adaptive change in STRA6 by competition between species.

## Supporting Information

File S1 **The information of STRA6 genes used in this study.**
(DOC)

File S2 **Alignment of all STRA6 amino acids analysed in Fasta format.**
(FASTA)

File S3 **Results of branch-site model A tests for detection of positively selected sites in selected branches.** ^a^ Log-likelihood value,^b^ k estimate of transition/transversion rate ratio,^c^ Positive selection sites are identified at the cutoff p>95%, with those with 99% shown in boldface.
(DOC)
