# Supplementary material for: Adaptive Evolution of the STRA6 Genes in Mammalian
Source: PLoS One. 2014 Sep 24;9(9):e108388. doi: 10.1371/journal.pone.0108388 (PMC4177561; doi:10.1371/journal.pone.0108388)
Supplement: File S1 — The information of STRA6 genes used in this study. (DOC) [file pone.0108388.s001.doc]

**Supporting file 1. The information of STRA6 genes used in this study.**

| **Order** | **Species name in English** | **Species name in Latin** | **Sequences Source** |
| --- | --- | --- | --- |
| *Afrosoricida* | Lesser hedgehog tenrec | *Echinops telfairi* | *XM 004712031* |
| *Afrosoricida* | Cape golden mole | *Chrysochloris asiatica* | *XM 006866569* |
| *Anseriformes* | Mallard | *Anas platyrhynchos* | *XM 005021340* |
| *Anura* | Toad | *Xenopus tropicalis* | *XM_002934548* |
| *Artiodactyla* | Cow | *Bos taurus* | *NM_001075730* |
| *Artiodactyla* | Cashmere goat | *Capra hircus* | *http://goat.kiz.ac.cn* |
| *Artiodactyla* | Sheep | *Ovis aries* | *XM_004018101* |
| *Artiodactyla* | Pig | *Sus scrofa* | *ENSSSCG00000001909* |
| *Artiodactyla* | Alpaca | *Vicugna pacos* | *XM 006213725* |
| *Artiodactyla* | Wild Bactrian camel | *Camelus ferus* | *XM 006182032* |
| *Artiodactyla* | chiru | *Pantholops hodgsonii* | *XM 005962280* |
| *Artiodactyla* | Water buffalo | *Bubalus bubalis* | *XM 006047354* |
| *Carnivora* | Dog | *Canis familiaris* | *XM_849193* |
| *Carnivora* | Cat | *Felis catus* | *XM_003986970* |
| *Carnivora* | Panda | *Ailuropoda melanoleuca* | *ENSAMEG00000009123* |
| *Carnivora* | Ferret | *Mustela putorius furo* | *ENSMPUG00000017269* |
| [*Carnivora*](http://en.wikipedia.org/wiki/Carnivora) | Walrus | *Odobenus rosmarus* | *XM 004407810* |
| [*Carnivora*](http://en.wikipedia.org/wiki/Carnivora) | Siberian tiger | *Panthera tigris altaica* | *XM 007084680* |
| *Cetacea* | Bottlenosed dolphin | *Tursiops truncatus* | *XM_004314357* |
| *Cetacea* | Common minke whale | *Balaenoptera acutorostrata scammoni* | *XM 007197727* |
| *Cetacea* | Sperm whale | *Physeter catodon* | *XM 007110834* |
| *Cetacea* | Baiji | *Lipotes vexillifer* | *XM 007446667* |
| *Cetacea* | [Killer whale](http://en.wikipedia.org/wiki/Killer_whale) | *Orcinus orca* | *XM 004276293* |
| *Chimaeriformes* | Australian ghostshark | *Callorhinchus milii* | *XM 007908079* |
| *Chiroptera* | Megabat | *Pteropus vampyrus* | *ENSPVAG00000003862* |
| *Chiroptera* | Microbat | *Myotis lucifugus* | *ENSMLUG00000002427* |
| *Chiroptera* | Big brown bat | *Eptesicus fuscus* | *XM_008157086* |
| *Chiroptera* | Mouse-eared bats | *Myotis davidii* | *XM 006753250* |
| *Chiroptera* | Black flying fox | *Pteropus alecto* | *XM 006922634* |
| *Cingulata* | Nine-banded armadillo | *Dasypus novemcinctus* | *XM_004476621* |
| *Coelacanthiformes* | Coelacanth | *Latimeria menadoensis* | *ENSLACG00000005870* |
| *Columbiformes* | Rock dove | *Columba livia* | *XM 005513040* |
| *Crocodilia* | American alligator | *Alligator mississippiensis* | *XM 006273156* |
| *Crocodilia* | Chinese alligator | *Alligator sinensis* | *XM 006020331* |
| *Cypriniformes* | Zebrafish | *Danio rerio* | *NM_001045312* |
| *Cyprinodontiformes* | Platyfish | *Xiphophorus maculatus* | *ENSXMAG00000010292* |
| *Eulipotyphla* | Common shrew | *Sorex_araneus* | *XM 004611839* |
| *Eulipotyphla* | European hedgehog | *Erinaceus_europaeus* | *XM_007536992* |
| *Eulipotyphla* | Star-nosed mole | *Condylura cristata* | *XM 004687574* |
| *Falconiformes* | Saker falcon | *Falco cherrug* | *XM 005437459* |
| *Falconiformes* | Peregrine falcon | *Falco peregrinus* | *XM 005232566* |
| *Galliformes* | Turkey | *Meleagris gallopavo* | *ENSMGAG00000002915* |
| *Galliformes* | Chicken | *Gallus gallus* | *XM_413689* |
| *Gasterosteiformes* | Stickleback | *Gasterosteus aculeatus* | *ENSGACG00000017063* |
| *Lagomorpha* | Rabbit | *Oryctolagus cuniculus* | *ENSOCUG00000026358* |
| *Lagomorpha* | American pika | *Ochotona princeps* | *XM 004594635* |
| *Macroscelidea* | Elephant Shrews | *Elephantulus edwardii* | *XM 006888391* |
| *Marsupialia* | Opossum | *Monodelphis domestica guttatus* | *ENSMODG00000009743* |
| *Monotremata* | Platypus | *Ornithorhynchus anatinus* | *XM 007664717* |
| *Passeriformes* | Medium ground finch | *Geospiza fortis* | *XM 005430052* |
| *Passeriformes* | Collared flycatcher | *Ficedula albicollis* | *XM 005051599* |
| *Perciformes* | Tilapia | *Oreochromis niloticus* | *ENSONIG00000015383* |
| *Perciformes* | Bicolor damselfish | *Stegastes partitus* | *XM 008276819* |
| *Perissodactyla* | Horse | *Equus caballus* | *ENSECAG00000016969* |
| *Perissodactyla* | White rhinoceros | *Ceratotherium simum simum* | *XM_004421802* |
| *Petromyzoniformes* | Lamprey | *Petromyzon marinus* | *ENSPMAG00000002512* |
| *Primates* | Human | *Homo sapiens* | *NM_001142617* |
| *Primates* | Bonobo | *Pan_paniscus* | *XM_003811116* |
| *Primates* | Green monkey | *Chlorocebus sabaeus* | *XM_008015906* |
| *Primates* | Bushbaby | *Galago senegalensis* | *ENSOGAT00000032530* |
| *Primates* | Orangutan | *Pongo pygmaeus abelii* | *NM_001133670* |
| *Primates* | *Pygmy chimpanzee* | *Pan paniscus* | *XM_003811116* |
| *Primates* | Small-eared galago | *Otolemur garnettii* | *XM_003784670* |
| *Primates* | Olive baboon | *Papio anubis* | *XM_003901189* |
| *Primates* | Chimpanzee | *Pan troglodytes* | *XM_510665* |
| *Primates* | Western gorilla | *Gorilla gorilla* | *XM_004056496* |
| *Primates* | Rhesus macaque | *Macaca mulatta* | *XM_001098728* |
| *Proboscidea* | Elephant | *Loxodonta africana* | *ENSLAFG00000011053* |
| *Psittaciformes* | Budgerigar | *Melopsittacus undulatus* | *XM 005145778* |
| *Rodentia* | House mouse | *Mus musculus* | *NM_001162475* |
| *Rodentia* | Guinea pig | *Cavia porcellus* | *ENSCPOG00000026259* |
| *Rodentia* | Rat | *Rattus norvegicus* | *NM_001029924* |
| *Rodentia* | Long-tailed chinchilla | *Chinchilla lanigera* | *XM 005393572* |
| *Rodentia* | Degu | *Octodon degus* | *XM 004628902* |
| *Rodentia* | Lesser Egyptian jerboa | *Jaculus jaculus* | *XM 004666643* |
| *Rodentia* | Deer mouse | *Peromyscus maniculatus bairdii* | *XM 006971507* |
| *Rodentia* | Prairie vole | *Microtus ochrogaster* | *XM 005369467* |
| *Rodentia* | Chinese hamster | *Cricetulus griseus* | *XM 007643010* |
| *Rodentia* | Golden hamster | *Mesocricetus auratus* | *XM 005069606* |
| *Rodentia* | Naked mole rat | *Heterocephalus glaber* | *XM_004898744* |
| *Scandentia* | Treeshrew | *Tupaia chinensis* | *XM 006153377* |
| *Sirenia* | West Indian Manatee | *Trichechus manatus latirostris* | *XM 004374740* |
| *Squamata* | Burmese python | *Python bivittatus* | *XM 007430096* |
| *Testudines* | Chinese softshell turtle | *Pelodiscus sinensis* | *ENSPSIG00000011312* |
| *Testudines* | Green sea turtle | *Chelonia mydas* | *XM 007059906* |
| *Testudines* | Painted turtle | *Chrysemys picta bellii* | *XM 005290507* |
| *Tetraodontiformes* | Tetraodon | *Tetraodon nigroviridis* | *ENSTNIG00000005151* |
| *Tetraodontiformes* | Fugu | *Takifugu rubripes* | *ENSTRUG00000015637* |
| *Tubulidentata* | Aardvark | *Orycteropus afer* | *XM 007949001* |
