# Supplementary material for: Adaptive Evolution of the STRA6 Genes in Mammalian
Source: PLoS One. 2014 Sep 24;9(9):e108388. doi: 10.1371/journal.pone.0108388 (PMC4177561; doi:10.1371/journal.pone.0108388)
Supplement: File S3 — Results of branch-site model A tests for detection of positively selected sites in selected branches. a Log-likelihood value,b k estimate of transition/transversion rate ratio,c Positive selection sites are identified at the cutoff p>95%, with those with 99% shown in boldface. (DOC) [file pone.0108388.s003.doc]

**Additional file 3. Results of branch-site model A tests for detection of positively selected sites in selected branches.**

| **Model** | **np^a^** | ***Parameters*** | ***L^b^*** | ***p*** | **Positively selected sites ^c^** |
| --- | --- | --- | --- | --- | --- |
| Null hypothesis for branch Primates | *179* | *proportion 0.66031 0.32912 0.00705 0.00352*  *background w 0.17519 1.00000 0.17519 1.00000*  *foreground w 0.17519 1.00000 1.00000 1.00000* | *-74183.73* | 0 | Not allowed |
| Alternative hypothesis for Branch Primates | *180* | *proportion 0.64959 0.33077 0.01301 0.00663*  *background w 0.17589 1.00000 0.17589 1.00000*  *foreground w 0.17589 1.00000 78.80329 78.80329* | -74148.76 |  | 355 T |
| Null hypothesis for branch Rodent | *179* | *proportion 0.60211 0.29404 0.06977 0.03408*  *background w 0.16832 1.00000 0.16832 1.00000*  *foreground w 0.16832 1.00000 1.00000 1.00000* | *--74132.92* | 1 | Not allowed |
| Alternative hypothesis for Branch Rodent | *180* | *proportion 0.60210 0.29404 0.06978 0.03408*  *background w 0.16832 1.00000 0.16832 1.00000*  *foreground w 0.16832 1.00000 1.00000 1.00000* | *-74132.92* |  | 92 S **135 V 183 G**  **220 D 517 Y 578 I 801 V** 820 A |
| Null hypothesis for Branch Carnivora | *179* | *proportion 0.63409 0.31331 0.03521 0.01740*  *background w 0.17384 1.00000 0.17384 1.00000*  *foreground w 0.17384 1.00000 1.00000 1.00000* | *--74179.69* | 0 | Not allowed |
| Alternative hypothesis for Branch Carnivora | *180* | *proportion 0.63544 0.29895 0.04462 0.02099*  *background w 0.17632 1.00000 0.17632 1.00000*  *foreground w 0.17632 1.00000 998.79000 998.79000* | -74127.79 |  | 182 P 986 - |
| Null hypothesis for branch Chiroptera | *179* | *proportion 0.65036 0.32393 0.01716 0.00855*  *background w 0.17501 1.00000 0.17501 1.00000*  *foreground w 0.17501 1.00000 1.00000 1.00000* | -74183.43 | 0.0000 | Not allowed |
| Alternative hypothesis for Branch Chiroptera | *180* | *proportion 0.65070 0.32637 0.01527 0.00766*  *background w 0.17556 1.00000 0.17556 1.00000*  *foreground w 0.17556 1.00000 9.53455 9.53455* | *-74172.62* |  | 260 D **261 G**  **262 K** 896 E |
| Null hypothesis for Branch Cetartiodactyla | *179* | *proportion 0.61889 0.30062 0.05418 0.02632*  *background w 0.17276 1.00000 0.17276 1.00000*  *foreground w 0.17276 1.00000 1.00000 1.00000* | *--74167.44* | 0.0494 | Not allowed |
| Alternative hypothesis for Branch Cetartiodactyla | *180* | *proportion 0.63534 0.30775 0.03834 0.01857*  *background w 0.17335 1.00000 0.17335 1.00000*  *foreground w 0.17335 1.00000 1.63308 1.63308* | *-74165.51* |  | 187 P  **668 A**  752 A  **790 Y** **863 M** |
| Null hypothesis for branch Sauropsida | *179* | *proportion 0.60637 0.28102 0.07695 0.03566*  *background w 0.16733 1.00000 0.16733 1.00000*  *foreground w 0.16733 1.00000 1.00000 1.00000* | --74102.53 | 1 | Not allowed |
| Alternative hypothesis for Branch Sauropsida | *180* | *proportion 0.60637 0.28102 0.07695 0.03566*  *background w 0.16733 1.00000 0.16733 1.00000*  *foreground w 0.16733 1.00000 1.00000 1.00000* | *-74102.53* |  | **91 M** **98 N** **99 Q**  **100 T**  **107 D** 111 Y  **244 E**  **252 L** **339 A**  **352 L** **383** L 394 G 445 L 493 L  **514 S 556 A 616 E**  **655 V** 717 T |
| Null hypothesis for branch *Actinopterygii* | *179* | *proportion 0.57295 0.29157 0.08979 0.04569*  *background w 0.16750 1.00000 0.16750 1.00000*  *foreground w 0.16750 1.00000 1.00000 1.00000* | *--74121.98* | 1 | Not allowed |
| Alternative hypothesis for Branch *Actinopterygii* | *180* | *proportion 0.57296 0.29154 0.08980 0.04569*  *background w 0.16750 1.00000 0.16750 1.00000*  *foreground w 0.16750 1.00000 1.00000 1.00000* | -74121.98 |  | **131 P** 171 R **242 P 507 C 521 K 523 G 526 S 528 A 556 A**  562 A **577 T 580 K 583 A 597 G 599 V**  639 C **644 L**  732 R  **754 A**  **889 Q** |
| Null hypothesis for branch *Afrotheria* | *179* | *proportion 0.60826 0.29424 0.06571 0.03179*  *background w 0.17169 1.00000 0.17169 1.00000*  *foreground w 0.17169 1.00000 1.00000 1.00000* | -74160.45 | 0.0215 | Not allowed |
| Alternative hypothesis for Branch *Afrotheria* | *180* | *proportion 0.62858 0.30207 0.04684 0.02251*  *background w 0.17304 1.00000 0.17304 1.00000*  *foreground w 0.17304 1.00000 1.97702 1.97702* | -74157.81 |  | **136 P 140 T 316 - 355 T**  523 G 960 A |

*Note:*

*^a^ Log-likelihood value*

*^b^ k estimate of transition/transversion rate ratio*

*^c^ Positive selection sites are identified at the cutoff p>90%, with those with 95% shown in boldface.*
